# An artificial intelligence system applied to recurrent cytogenetic aberrations and genetic progression scores predicts *MYC* rearrangements in large B‐cell lymphoma

**DOI:** 10.1002/jha2.451

**Published:** 2022-05-16

**Authors:** Rolando García, Anas Hussain, Weina Chen, Kathleen Wilson, Prasad Koduru

**Affiliations:** ^1^ Department of Pathology UT Southwestern Medical Center Dallas Texas USA; ^2^ Deccan College of Medical Sciences Hyderabad India

**Keywords:** AI, chromosome aberrations, DLBCL, genetic progression scores, MYC rearrangement

## Abstract

Diffuse large B‐cell lymphoma (DLBCL), the most common type of non‐Hodgkin lymphoma, is characterized by *MYC* rearrangements (*MYC* R) in up to 15% of cases, and these have unfavorable prognosis. Due to cryptic rearrangements and variations in *MYC* breakpoints, *MYC* R may be undetectable by conventional methods in up to 10%–15% of cases. In this study, a retrospective proof of concept study, we sought to identify recurrent cytogenetic aberrations (RCAs), generate genetic progression scores (GP) from RCAs and apply these to an artificial intelligence (AI) algorithm to predict *MYC* status in the karyotypes of published cases. The developed AI algorithm is validated for its performance on our institutional cases. In addition, cytogenetic evolution pattern and clinical impact of RCAs was performed. Chromosome losses were associated with *MYC*‐, while partial gain of chromosome 1 was significant in *MYC* R tumors. *MYC* R was the sole driver alteration in *MYC*‐rearranged tumors, and evolution patterns revealed RCAs associated with gene expression signatures. A higher GPS value was associated with *MYC* R tumors. A subsequent AI algorithm (composed of RCAs + GPS) obtained a sensitivity of 91.4 and specificity of 93.8 at predicting *MYC* R. Analysis of an additional 59 institutional cases with the AI algorithm showed a sensitivity and specificity of 100% and 87% each with positive predictive value of 92%, and a negative predictive value of 100%. Cases with a *MYC* R showed a shorter survival.

## 1 INTRODUCTION

**FIGURE 1:**
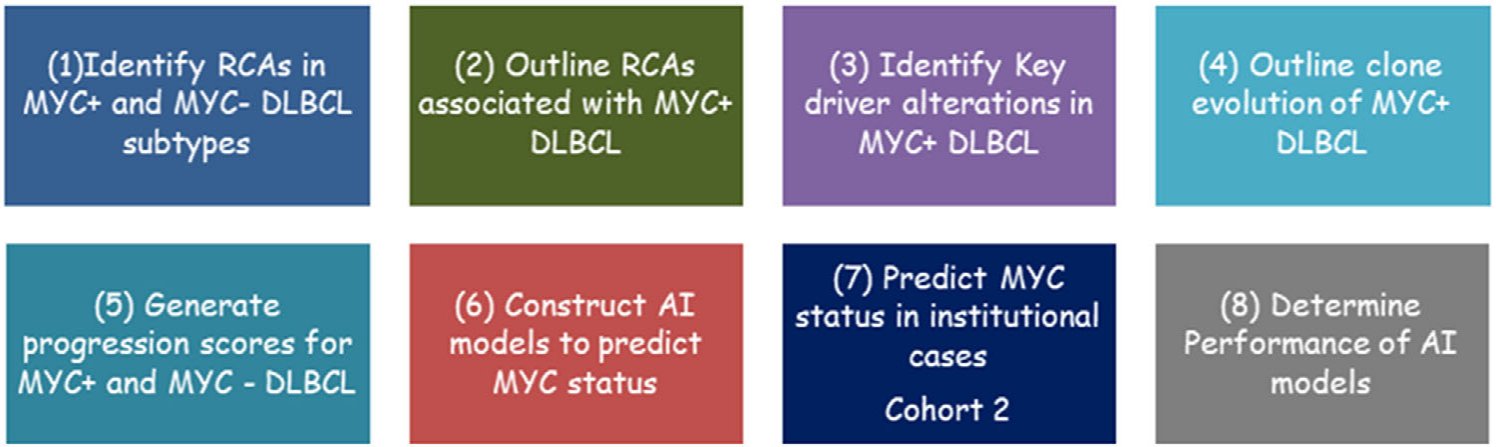
General objectives of the study

Diffuse large B‐cell lymphoma (DLBCL) is the most common histological subtype of non‐Hodgkin lymphoma (NHL) comprising greater than 30% of NHL cases [1]. It is a heterogeneous disease with different clinical, histological, and molecular features. Up to 10%–15% of these cases carry a *MYC* rearrangement at chromosome band region 8q24 [2]. Rearrangements involving *MYC* result in a deregulated expression of MYC due to the juxtaposition of transcriptional enhancer elements of the immunoglobulin (*IG*) genes with *MYC*. Such events lead to the overexpression of MYC that is considered to play a pivotal role in the pathogenesis of the disease [3]. In a small number of cases, *MYC* R may include non*‐IG* genes [4]. The most notable translocations involving *MYC* and *IG* loci in DLBCL include t(8;14)(q24;q32) leading to a *MYC* and *IG* heavy chain fusion (*MYC‐IGH*), t(8;22)(q24;q11) resulting in a *MYC‐IGL* (lamda light chain) fusion and the less common, t(2;8)(p12;q24) that results in a *MYC‐IGK* (kappa light chain) fusion with frequencies of 70%, 22%, and 8% respectively [5, 6]. In a small number of cases, *MYC* R may include non*‐IG* genes [4]. In terms of clinical outcome, DLBCL with *MYC* R (herein after designated as *MYC*+) has a decreased survival compared to other chromosome aberrations or those lacking a *MYC* R (herein after designated as *MYC−*); these cases may require more aggressive therapeutic regimens than the rituximab plus cyclophosphamide, doxorubicin, vincristine, and prednisolone (R‐CHOP) [1, 7–12]. Preliminary studies have indicated a positive prognosis in *MYC*+ patients on aggressive treatment [13, 14]. Therefore, establishing a *MYC* status in these patients is essential for prognostic purposes. Due to cryptic rearrangements and variation in *MYC* breakpoints, both chromosome and fluorescence in situ hybridization (FISH) analysis may fail to detect these translocations in some cases [15, 16, 17]. In case of FISH analysis, up to 10% of the cases may be incorrectly identified [18, 19, 20, 21]. Indeed, Haralambieva et al. [21] reported 11% of *MYC* breakpoints may lie far from the 5′ or 3′ end of the *MYC* itself. In a separate study, 8q24 breakpoints were mapped greater than 350–645 kb 3′‐downstream from *MYC* inside a cluster region [22]. Consequently, current commercially available FISH probes such as the dual color dual fusion probe set and the *MYC* break‐apart probe may both fail to detect *MYC* R. Furthermore, other genetic alterations such as mutations, cryptic insertion of *MYC* into *IGH*, cryptic insertion of *IG* regulatory regions into *MYC*, deregulation of micro RNA‐34B, or single nucleotide polymorphisms at 8q24 that may convey a shared underlying biology to *MYC* R have been implicated [15]. In fact, Hilton et al. [23] showed that the expression signature of *MYC* high grade DLBCL in which *MYC* had either cryptic alterations or rearrangements with non‐*IG* partners is similar to the *MYC* double‐hit DLBCL. Considering this and because of the clinical impact of *MYC* R, we sought to develop artificial intelligence (AI) systems composed of recurrent cytogenetic aberrations (RCAs) and derived genetic progression score (GPS) to predict *MYC*+ DLBCL tumors. In addition, we also performed identification of driver versus passenger alterations, evolution patterns in *MYC*+ tumors, and the clinical impact of RCAs on patient survival (Figure 1).

### 1.1 The dataset and analysis methods

Mitelman Database of Chromosome Aberrations and Gene Fusions in Cancer (https://mitelmandatabase.isb‐cgc.org, accessed on 5/20/2020) was searched for DLBCL cases during 1983–2019. This list was curated for cases with a break at 8q24 to identify *MYC*‐rearranged (classical and nonclassical) and cases with no rearrangement at 8q24; these constituted cohort 1 cases. Initially, karyotypes were evaluated using CytoGPS [24], a software tool to parse karyotype nomenclature to identiy RCAs. Thereafter, each case was curated manually. A Fisher Exact two‐tail test, a chi‐square test, and a Bonferroni adjusted *p*‐value were used to identify differences between the two groups. The Translational oncology package (TRONCO) in the R‐environment was used to map evolutionary trajectory of RCAs [25].

**FIGURE 2:**
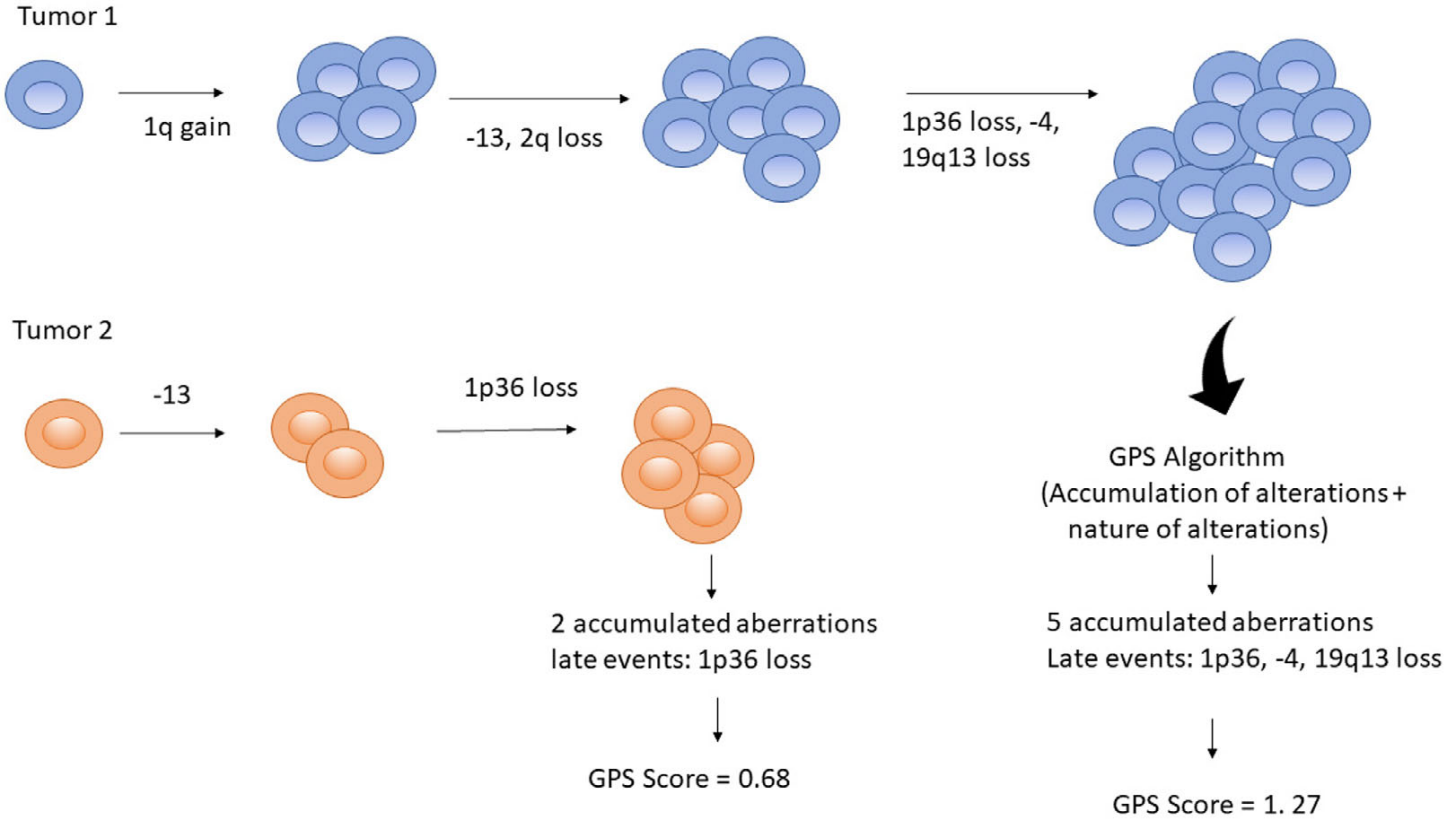
The schematic illustrates the generation of genetic progression score (GPS) based on the number of accumulated aberrations and time of occurrence of the aberrations from a computed temporal oncogenic tree or trajectory pathway (i.e., late event vs. early event) [27]. A late event obtains a higher weighted value than an earlier event, for example, 1p36 loss is assigned a higher value than −13; thus, higher number of accumulated aberrations and late events receive a higher score

Rtreemix package was used to calculate GPS [27]. The GPS is derived from the number or accumulation of genetic aberrations and the types of the aberrations from the data set. Late events that developed during tumor progression receive a higher weighted value compared to early events. Thereafter, the weighted value of each RCA is used to calculate the GPS of each tumor. A higher score suggests a higher‐grade tumor with adverse outcome (Figure 2).

**FIGURE 3:**
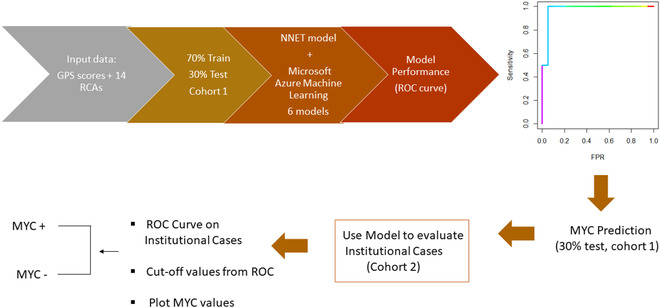
The general workflow of the *MYC* prediction model.

The GPS for each tumor was then combined with RCAs to develop the AI algorithm. The system was composed of a neural network with 15 inputs and one output. A 10‐cross validation was applied, and the neutral network (NNET) package was used to build the algorithm [28]. The NNET was selected because of its flexibility to outline each of the cases as *MYC*+ or *MYC*− based on a threshold value from the receiver operating characteristic (ROC) curve, as opposed to the “black box” prediction from the other classifiers. ROC curve was performed to evaluate the discrimination ability of the system. Seventy percent of cases from cohort 1 were used to train the system, and the remaining 30% of cases were used to test the system to predict *MYC* status. The tested NNET AI algorithm was validated on 59 institutional cases (cohort 2, approved by the institutional review board (IRB)) to predict *MYC* status (Figure 3). Six additional AI algorithms—GBoost, MaxAbsScaler/ Light GBM, Support Vector Machine ‐ SVM, Random Forest Tree, SparseNormalizer KCNN, and Standard Scan Wrapper Logistic Regression from the open‐source Microsoft Azure Machine Learning Platform (http://azure.microsoft.com)—were also used to predict the *MYC* status and compared the outcome with that of NNET AI algorithm.

### 1.2 Cohort 2 cases

All cases of high grade DLBCL with karyotype and FISH ascertained during 2005–2020 (31 *MYC*+: 13 bone marrow, seven lymph nodes, nine other tumor site; 28 *MYC*−: eight bone marrow, 11 lymph nodes, eight other tumor site, tumor site not available for three cases) were included in the study. Fresh clinical specimens obtained at diagnosis were processed into tissue culture within 4 h of collection and were evaluated for G‐banded karyotype and for *MYC* status using *MYC*/*IGH* dual colored dual fusion probe and by *MYC* break‐apart probe (Abbott, Abbott Park, Illinois, USA). Karyotypes were prepared from G‐banded metaphases present in short term cultures (24 h) using standard protocols. FISH was performed on slides prepared from cultured specimen or on touch preparations of tissues using probes described above using standard protocols.

## 2 RESULTS

**FIGURE 4:**
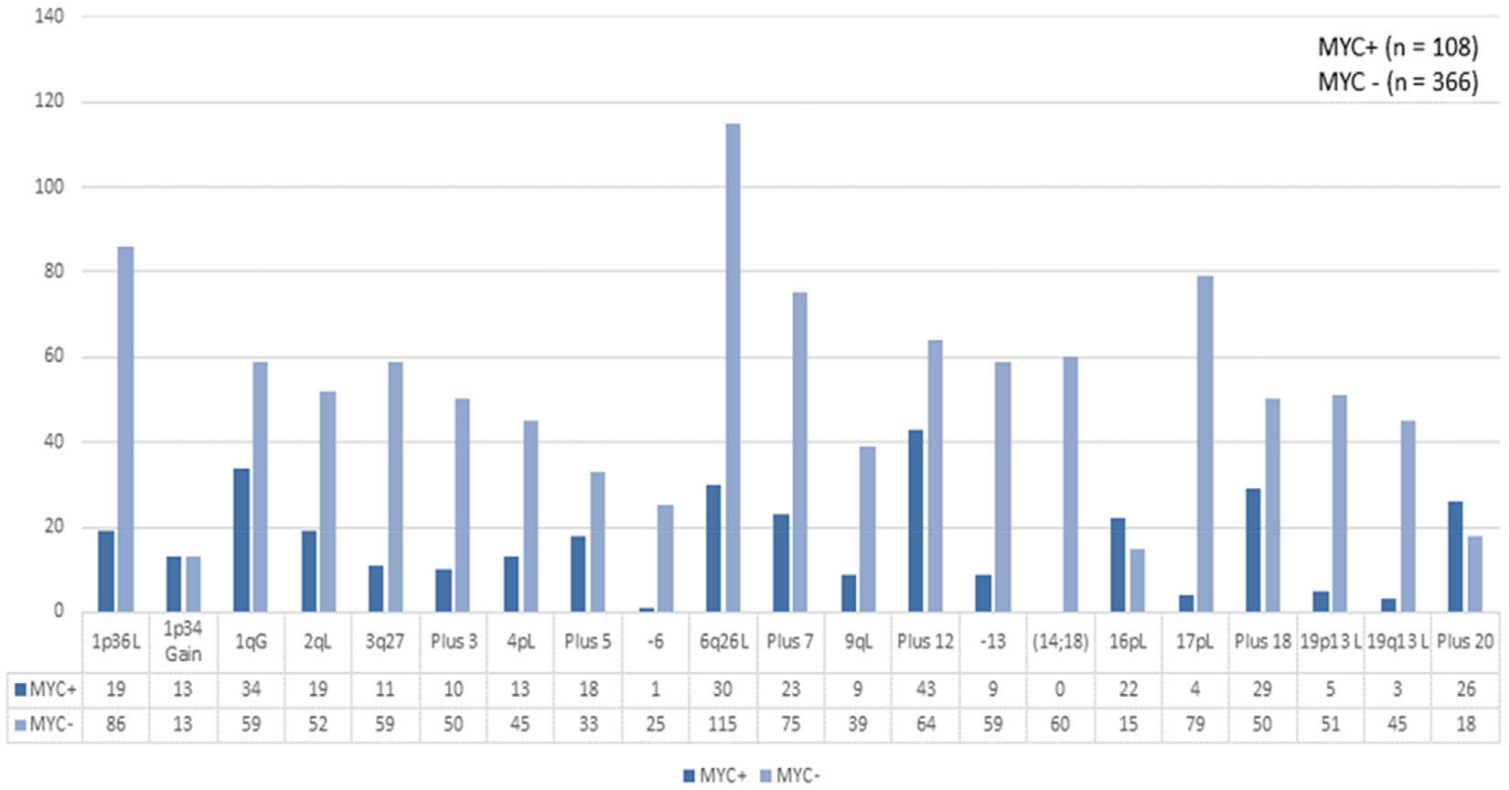
Recurrent cytogenetic aberrations (RCAs) and the total number of events from cohort 1 tumors. Key: L, loss; G, gain; p, short arm of a chromosome; q, long arm of a chromosome

A total of 474 cases of DLBCL (108 *MYC*+, 366 *MYC*−) were retrieved from the Mitelman database (cohort 1). Majority of cases (80%) had a classical t(8;14)(q24;q32) followed by t(8;22)(q24;q11) in 11%, t(2;8)(p12;q24) in 2%; six of these had cryptic *MYC* R due to complex chromosomal rearrangements involving MYC IG. MYC/non‐IG rearrangements ([t(8;9)(q24;p13)], *n* = 2; del(8)(q24), *n* = 3; [t(3;8)(q27;q24)], *n* = 2; [t(4;8)(q21,q33;q24)], *n* = 2; [t(7;8)(p12;q24)], *n* = 1; [t(3;8)(p24;q24)], *n* = 1; [t(8;18)(q24;p11], *n* = 1; [t(8;16)(q24;p11), *n* = 1; inv(8)(p21;q24), *n* = 1) were present in 13% of cases. Manual curation of karyotypes from these cases generated 22 RCAs (Figure 4).

**FIGURE 5:**
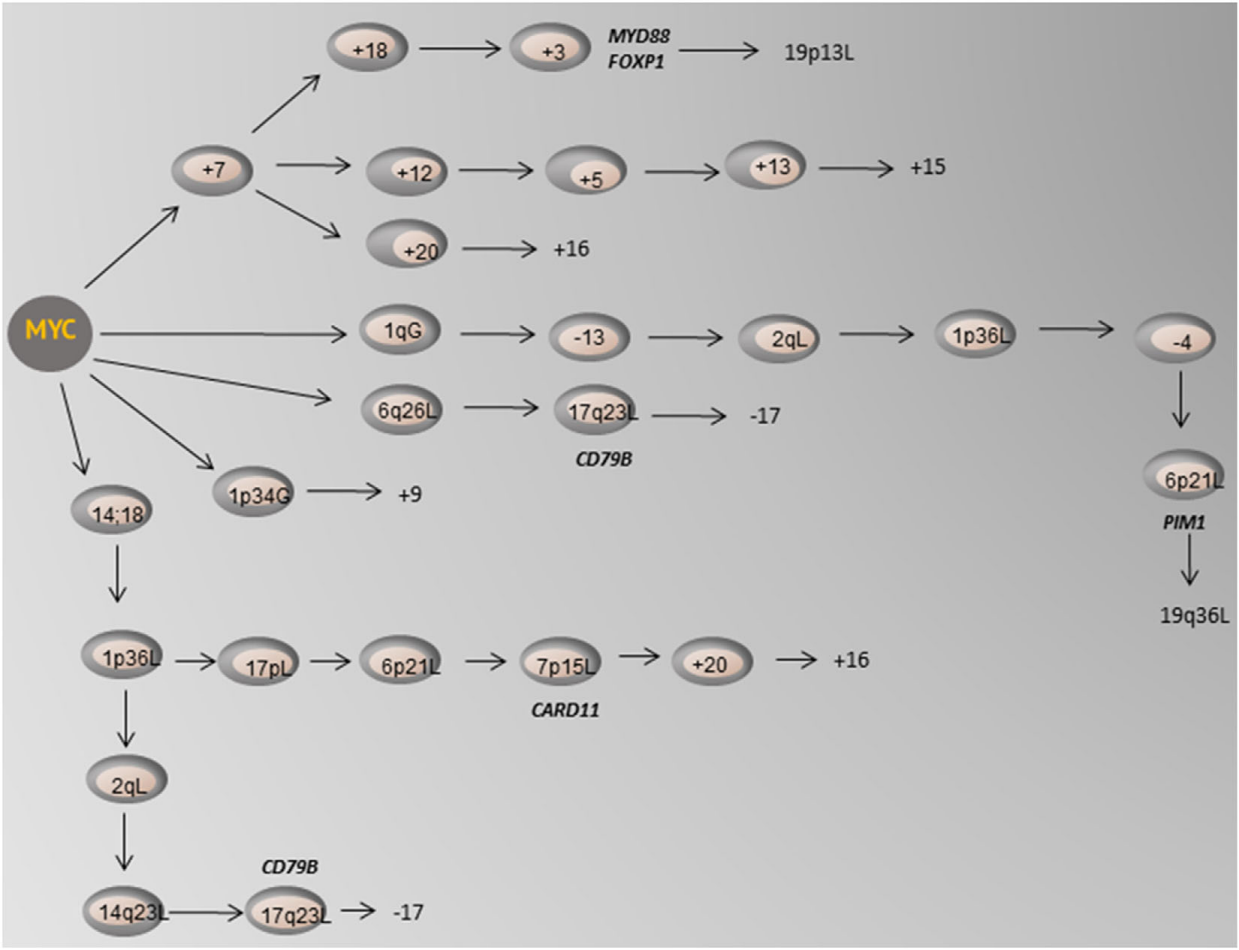
Evolution patterns in *MYC*+ diffuse large B‐cell lymphoma (DLBCL) detected by TRONCO. Structural and numerical aberrations related to gene expression profiles of *MYC*+ DLBCL include trisomy of chromosome 3 resulting in altered gene expression of *MYD88* and *FOXP1* and 17q23 and 7p15 loss leading to gene expression changes in *CD79B* and *CARD11*, respectively, and 6p21 loss resulting in altered *PIM1* gene expression. Trisomy of chromosomes 9, 15, 16, 20, and loss of chromosome 17, and loss at 19p13 and 19q13 appeared late in tumor progression

Of these RCAs, gain of 1p34 and 1q14 was significantly associated with *MYC* tumors (*p* = 0.003 and *p* = 0.0008 respectively), whereas losses of chromosomes were associated with *MYC*− tumors (141 *MYC*+ vs. 611 *MYC*− *p* < 0.001). In *MYC*+ tumors, a *MYC* R was the single driver alteration, and evolution patterns revealed RCAs associated with reported gene expression profiles in *MYC*+ DLBCL, mainly *FOXP1*, *MYD88*, *CD79B*, *PIM1*, and *CARD11* (Figure 5).

**FIGURE 6:**
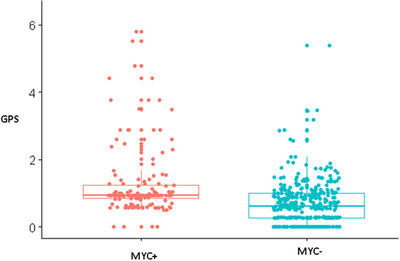
Genetic progression scores generated from 14 recurrent cytogenetic aberrations (RCAs) for *MYC* and *MYC*− tumors; difference in genetic progression score between these groups was significant (*p* < 0.0001)

To generate GPS, only 14 RCAs were processed due to the large amount of memory needed to perform this computation. RCAs included in this analysis were gains of chromosomes 1p34, 1q14, 5, 7, 12, and 18, and loss of 1p36, 2q, 4p, 13q, 17p, 19p13, as well as t(14;18). These were then applied to the Rtreemix algorithm to generate a GPS for each tumor. Generated scores showed a significant difference between *MYC* versus *MYC*− tumors with an average value of 1.27 versus 0.68 each (*p* < 0.0001) (Figure 6).

**FIGURE 7:**
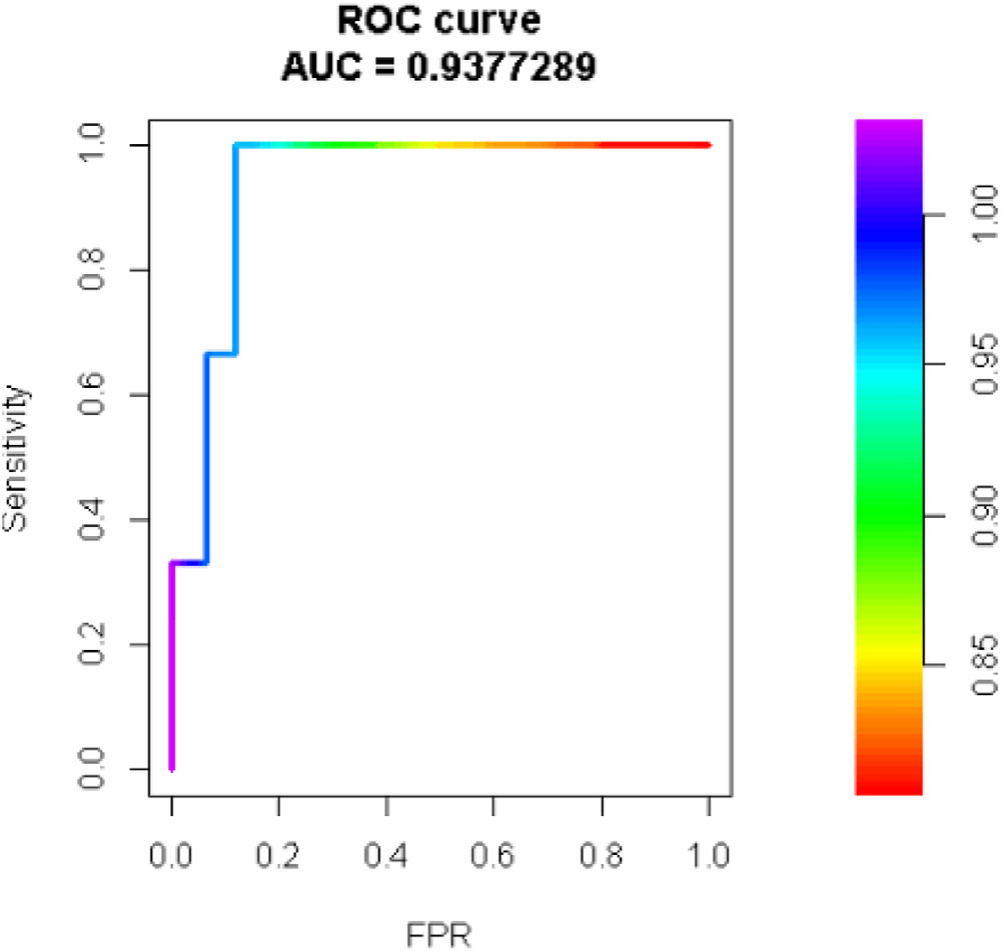
Receiver operating characteristic curve of the NNET artificial intelligence (AI) system composed of 14 recurrent cytogenetic aberrations and genetic progression scores. The area under the receiver operating characteristic showed a diagnostic ability of 93.8% at predicting a *MYC*+ in diffuse large B‐cell lymphoma (DLBCL). FPR, false positive rate

The generated GPSs were then combined with RCAs to develop an AI system for detecting *MYC* R. The system was trained on 332 cases and tested on 142 cases from cohort 1. An ROC curve showed an area under the curve (AUC) of 93.8% with a sensitivity of 91.4 and specificity of 93.8 at predicting *MYC* R (Figure 7).

**FIGURE 8:**
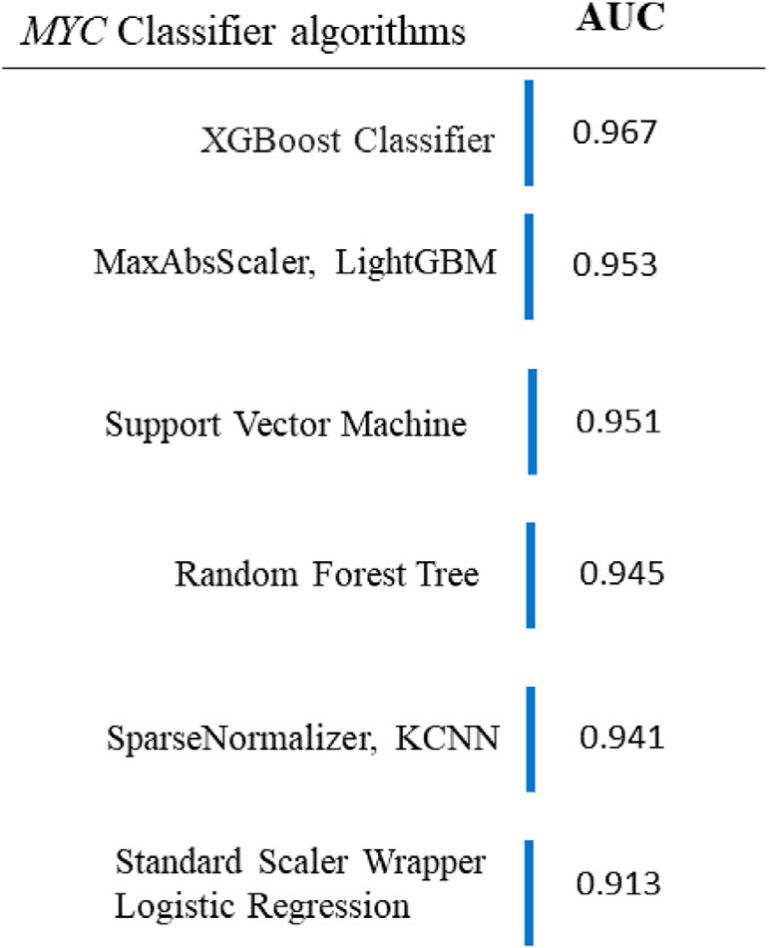
*MYC* classifiers from the Azure artificial intelligence (AI) platform. The area under the curve (AUC) describes the diagnostic ability to predict *MYC* rearrangement by different additional classifiers

Additional six classifiers using the open‐source Microsoft Azure Machine Learning Studio obtained similar results to the NNET model. Indeed, five of the six classifiers outperformed the NNET model (Figure 8).

Using these classifiers, GPS was the most important feature predictor of a *MYC* R.

The 59 institutional cases (Table 1) were used to test the capability of the RCA‐GPS NNET AI model to predict *MYC* status and to evaluate the clinical impact of RCAs on patient outcome.

**TABLE 1:** Clinical features and karyotypes of the 59 institutional cases (cohort 2)

ID	Age–gender	Tumor site	Treatment	Follow‐up	Status	Karyotype
1	44 M	Lymph node	NA	NA	NA	47, XY, +X, del(1)(p31), dup(2)(p11.2p25), **t(8;14)(q24;q32)**, del(15)(q13q15), add(16)(p13.1), t(14;18) (q32;q21)[15 cells]/46, XY [5 cells]
2	44 M	Thoracic spine mass	NA	NA	NA	47, XY, del(1)(p13.1p13.3), der(5)t(5;11)(q33;q13), +7, der(8)t(1;8)(q12;p23)**t(8;14)(q24;q32),** der(14)t(8;14)(q24;q32), del(16)(q11.1q24) [8 cells]/47, idem, ‐der(14)t(8;14)(q24;q32), +der(14)add(14) (p11.2)t(8;14)(q24;q32)[8 cells]/46, XY [4 cells]
3	36F	Bone marrow	CHOP	1	Expired	48∼49, XX, del(1)(p34.1p36.3), add(2)(p11.2), add(2)(q31), der(8)t(8;8)(p23;q11.2), der(8)**t(8;14)(q24.1;q32)**, add(9)(p22), der(12)t(1;12)(q21;p13)ins(12;?)(p13;?)add(12)(q24.1), add(13)(p11.2), add(14)(q32), der(14)add(14)(p11.2)t(8;14)(q24.1;q32), ‐15, ‐15, add(16)(q22), add(17)(p11.2), add(21)(q22), +3∼4 mar [cp4 cells]/46, XX [16 cells]
4	82 M	Left orbit	R‐CHOP	18	Expired	77 < 3n > , XXY, del(1)(p13p23), +2, t(3;9)(q21;p22), t(4;21)(q23;q11.2)?c, der(6)t(6;8)(q15;q22), +der(6)t(6;8)(q15;q22), psu idic(7)(q31), +i(7)(q10), ‐8, **t(8;14)(q24;q32)**, del(9)(p22p24), der(9)t(3;9) (q21;p22), +der(9)t(3;9)(q21;p22), ‐11, ‐15, +16, +20, +mar1, +3∼7mar.ish t(8;14)(MYC+, IGH+; MYC+, IGH+), der(6)(MYC+)[cp3]/46, XY, t(4;21)(q21;q11.2)?c[1]nuc ish (MYCx5, IGHx4)(MYC con IGHx2)[7/154]
5	40 M	Bone marrow	R‐hyper CVAD MTX and Ara‐c	5	Expired	49∼50, XY, +X, add(1)(q21), der(6)t(6;6)(q21;p12), **t(8;14)(q24.1;q32)**, +11, der(13)t(2;13)(q21;q34), der(15)t(1;15)(q12;q22), +17[cp2].nuc ish(MYC, IGH)x3(MYC con IGHx2)[22/100]
6	50F	Lymph node	N	NA	NA	49, XX, +X, del(3)(q12q21), dup(4)(q21q31), +7, der(8)**t(8;14)(q24;q32)**t(14;18)(q32;q21), der(14)t(8;14), der(18)t(14;18), +mar[15].ish der(8)(IGH+, BCL2+), der(14)(IGH+, BCL2‐), der(18) (IGH+, BCL2+)[15].nuc ish(5′MYCx3, 3′MYCx2)(5′MYC con 3′MYCx2)[104/200], (IGH, BCL2)x3(IGH con BCL2 × 1∼2)[162/200]
7	53 M	Bone marrow	R‐EPOCH	17	Expired	76∼81 < 3n > , XX, ‐Y, +X, del(1)(q32q41)x2, add(4)(q21), +6, +7, +add(7)(p11.2), der(8)add(8)(p21) **t(8;14)(q24.1;q32)**x2, add(9)(q13)x2, der(14)t(8;14)x2, add(15)(q24), add(17)(p13), add(17)(p11.2), +19, +20, der(22)t(11;22)(q13;q22)x2, +2∼3mar[cp2]/46, XY[15].nuc ish(MYC, IGH)x6(MYC con IGHx4)[1/200]
8	62 M	Abdominal mass	EPOCH	2	Expired	69∼87 < 4n > , XX, ‐Y, ‐Y, der(1)t(1;1)(p21;q21), ‐4, der(6;11)(p10;q10), **t(8;14)(q24.1;q32)**x2, +11, ‐12, ‐17, +20, ‐22[cp13]/85∼86, idem, +add(1)(p13)[cp2]
9	55F	Thyroid mass	Rituximab cytoxan, doxorubic vincristineetoposide Ara‐c.	75	Expired	48, XX, +3**, t(8;14)(q24.1;q32), +**12[cp10]/49, idem, t(3;5)(q27;q31), +der(5)t(3;5)[2]/46, XX[8].ish der(3)(5′BCL6+, 3′BCL6‐), der(5)(5′BCL6‐, 3′BCL6+), t(8;14)(MYC+, IGH+;MYC‐, IGH+)[cp7].nuc ish(5′BCL6, 3′BCL6)x2(5′BCL6 sep 3′BCL6 × 1)[4/200]/(5′BCL6, 3′BCL6)x3 (5′BCL6 sep 3′BCL6 × 1)[13/200]/(5′BCL6, 3′BCL6)x3(5′BCL6 con 3′BCL6 × 3)[95/200], (MYCx2, IGHx3)(MYC con IGHx1)[110/200], (IGHx3, BCL2 × 2)[116/200]
10	66F	NA	NA	NA	NA	46, XX, add(3)(q27), der(8)**t(8;14)(q24.1;q32)**t(14;18)(q32;q21), der(11)t(11;14)(q23;q32)t(8;14)t(14;18), der(14)t(8;14)t(14;18), der(16)t(1;16)(q12;q11.2), ‐17, der(18)t(14;18), ider(19)(q10) add(19)(q13.4), +mar[18].ish add(3)(5′BCL6‐, 3′BCL6+), der(8)(MYC+, IGH+, BCL2+), der(11) (MYC+, IGH+, BCL2+), der(14)(MYC+, IGH+, BCL2+), der(18)(IGH+, BCL2+), mar(5′BCL6++, 3′BCL6‐)[cp9].nuc ish(5′BCL6, 3′BCL6)x2(5′BCL6 con 3′BCL6 × 1)[59/200]/(5′BCL6 × 3, 3′BCL6 × 2)(5′BCL6 con 3′BCL6 × 1)[28/200], (MYCx4, IGHx4∼6) (MYC con IGHx2∼3)[67/200], (IGHx4∼6, BCL2 × 3∼4)(IGH con BCL2 × 2∼3)[93/200]
11	74 M	Pleural fluid	NA	NA	NA	48∼52, X, ‐Y, +1, +6, add(6)(q12)x2, +inv(7)(p11.2p22), del(8)(q13q22), +11, der(14)**t(8;14) (q24;q32)**t(14;18)(q32;q21), 15, add(17)(p13), add(16)(p13.1), der(18)t(14;18), +der(18)t(14;18)x2∼ 3[cp6]/49∼51, idem, ‐1, +13[cp4]/48∼49, idem, ‐1, ‐3, +add(3)(p12), ‐add(6), +6[cp6]/47∼48, idem, ‐1, ‐3, +der(3)add(3)(p21)add(3)(q21), ‐add(6), +6[cp4].ish del(8)(MYC+), der(14)t(8;14) t(14;18)(MYC+;IGH+)(IGH+;BCL2+)[cp4].nuc ish(5′BCL6, 3′BCL6)x3∼6(5′BCL6 con 3′BCL6 × 3∼6)[282/200], (MYCx2∼3, IGHx4∼7)(MYC con IGHx1)[196/200], (IGHx4∼8, BCL2 × 4∼7) (IGH con BCL2 × 3∼6)[199/200]
12	58 M	Lymph node	NA	NA	NA	50, XY, +3, +7, **t(8;14)(q24;q32)**, del(17)(p11.2p13), +18, +21[1].nuc ish(5′BCL6, 3′BCL6)x3(5′BCL6 con 3′BCL6 × 3)[47/200], (MYC, IGH)x3(MYC con IGHx2)[37/200], (IGH, BCL2)x3[42/200]
13	54 M	Bone marrow	DA‐EPOCH‐R	5	Expired	48, XY, inv(1)(p36.1q42), add(1)(q32), der(3)t(3;14)(q27;q11.2)**t(8;14)(q24;q32)**, del(6)(q13q21), +7, der(8)t(8;14), del(13)(q12q14), t(14;18)(q32;q21), +20[cp2]/46, XY[18].nuc ish(5′BCL6, 3′BCL6)x2 (5′BCL6 sep 3′BCL6 × 1)[2/200], (MYCx3, IGHx4)(MYC con IGHx2)[1/200], (5′MYC, 3′MYC)x2 (5′MYC sep 3′MYCx1)[1/200], (IGH, BCL2)x3(IGH con BCL2 × 2)[1/200]
14	47 M	Bone marrow	NA	1	Alive	46, XY, del(2)(p23p25), **t(8;14)(q24.1;q32**), add(12)(q24.1), add(13)(q34), del(17)(q24q25), del(18)(q21q23), add(20)(p13)[10]/46, XY[10].nuc ish(5′BCL6, 3′BCL6)x2(5′BCL6 con 3′BCL6 × 2)[200], (MYC, IGH)x3(MYC con IGHx2)[16/200], (5′MYC, 3′MYC)x2(5′MYC sep 3′MYCx1)[14/200], (IGH, BCL2)x2[200]
15	69F	Lymph node	NA	8	Expired	48, XX, +del(7)(q22q36), **t(8;14)(q24.1;q32), +**12[20]/46, XX[1].nuc ish(5′BCL6, 3′BCL6)x2(5′BCL6 con 3′BCL6 × 2)[200], (MYC, IGH)x3(MYC con IGHx2)[44/200], (IGHx3, BCL2 × 2)[34/200]
16	55F	Lung mass	DA‐R‐EPOCH; bortezomib with dose adjusted EPOCH	7	Alive	46, X, ‐X, der(1)del(1)(p12p22)(p36.1p36.3)t(1;14)(q21;q32)**t(8;14)(q24.1;q32)**, del(2)(p11.2p25), add(3)(q29), add(6)(p21), +del(6)(p21p23), add(7)(p22), der(8)t(8;14), t(9;11)(p13;q13), inv(12)(q22q24.1), der(14)t(8;14)t(1;14), der(16)t(1;16)(q12;q11.2), ‐17, ‐17, +18, +add(22)(q11.2)[cp12]/45, idem, ‐der(8), +ider(8)(q10)del(8)(q11.2q21)t(8;14), ‐18[cp8].ish der(1)(3′MYC+, IGH‐), der(8)(5′MYC+, IGH+), der(14)(3′MYC+, IGH+)[4].nuc ish(3′BCL6, 5′BCL6)x2 (3′BCL6 con 5′BCL6 × 2)[199], (MYC, IGH)x3∼4(MYC con IGHx1∼2)[176/200], (IGHx3∼4, BCL2 × 2∼3)[153/200]
17	55 M	NA	DA EPOCH + Velcade, Vidaza	5	Alive	43∼44, X, ‐Y, ‐1, add(1)(q12), add(3)(q21), ‐4, der(4)t(4;9)(q21;q13)?add(9)(q34), add(5)(q22), add(6)(p11), +7, der(8)**t(8;14)(q24;q32**), ‐9, add(9)(q34), ‐10, add(10)(p11.2), add(10)(q22), der(11)(11pter → 11q25::11q24 → 11q13::6p11 → 6pter), psu dic(14;1)(p12;q10)t(8;14), psu dic(15;1)(p12;q10), ‐18, der(18)t(1;18)(p32;q21), ‐20[cp11]/46, XY[9].nuc ish(CDKN2Cx1, CKS1Bx4)[31/200], (3′BCL6, 5′BCL6)x3(3′BCL6 con 5′BCL6 × 3)[14/200], (FGFR3 × 1, IGHx3∼4)[36/200], (5′MYC, 3′MYC)x2(5′MYC sep 3′MYCx1)[7/200], (5′MYCx3, 3′MYCx2)(5′MYC sep 3′MYCx1)[26/200], (MYC, IGH)x3(MYC con IGHx2)[15/200], (MYC, IGH)x4(MYC con IGHx3)[22/200], (D9Z1, D15Z4)x2[198], (CCND1 × 2, IGHx3∼4)[39/200], (RB1 × 2)[199], (IGHx3∼4, MAFx2)[33/200], (IGHx3∼4, BCL2 × 1)[36/200], (IGHx3∼4, MAFBx2)[34/200], (TP53 × 2)[194]
18	89 M	Bone marrow	NA	41	Expired	46, XY, del(2)(p11.2p13), del(6)(q23q27), add(7)(q32), t**(8;14)(q24.1;q32)**, add(17)(p11.2)[12]/46, idem, der(4)t(1;4)(q21;p14)[2]/46, XY[2].nuc ish(3′BCL6, 5′BCL6)x2(3′BCL6 con 5′BCL6 × 2)[28], (MYC, IGH)x3(MYC con IGHx2[12/36], (IGHx3, BCL2 × 2)[10/31] .46, XY, t(6;7)(q23;q36), t(8;14)(q24;q32), add(17)(p13)[2].nuc ish(3′BCL6, 5′BCL6)x2(3′BCL6 con 5′BCL6 × 2) [199], (D4Z1, D10Z1)x2[200], (RUNX1T1, RUNX1)x2[195], (MYC, IGH)x3(MYC con IGHx2)[50/200], (ABL1, BCR)x2[193], (5′KMT2A, 3′KMT2A)x2(5′KMT2A con 3′KMT2Ax2)[199], (ETV6, RUNX1)x2[199], (IGHx3, BCL2 × 2)[51/200]
19	70 M	Bone marrow	NA	7	Expired	47, XY, add(6)(p23), +7, add(8)(p23), **t(8;14)(q24.1;q32)**, der(13) t(13;15)(p12;q13), der(21)t(1;21)(q12;q22) [13]
20	57 M	Neck mass	CHOP‐R	4	Alive	80∼85 < 4n > ‐X, ‐Y, del(1)(p32p36.1), ‐2, ins(2;?)(q31;?)x2, ‐4, ‐4, ‐5, add(5)(p15), add(6)(q13), add(6)(q23), ins(6;?)(q23;?), add(7)(q11.2), **t(8;14)(q24;q32)**x2, add(9)(q22), add(9)(q22), ‐10, ‐10, ‐11, ‐12, ‐13, ‐15, +16, add(16)(p11.2), add(16)(p13.3), ‐18, ‐18, ‐20, ‐21, ‐22, ‐22, +7∼13mar[cp5]/75∼87, idem, add(19)(p13.3)[cp10]/46, XY[5]
21	66 M	Lymph node	CHOP‐R	18	Alive	51, X, ‐Y, +X, der(1)add(1)(p36.3)del(1)(q42q44), der(1)del(1)(p32p36.1)ins(1;?)(q21;?), add(2)(p11.2), der(2)t(2;7)(p21;q11.2), del(3)(p13p25), add(5)(q31), +der(5)t(5;14)(p14;q24)t(14;18)(q32;q21), +der(6)t(6;18)(q27;q21)t(14;18)(q32;q21), +7, add(8)(q24.1), ins(8;?)(q22;?), +add(10)(q22), +11, ‐13, der(13)t(13;14)(q32;q32), ‐14, der(14)**t(8;14)(q24;q32)**, der(16)t(7;16)(q11.2;p13.3), del(17) (p11.2p13), der(18)t(7;18)(q11.2;p11.2), +der(?)(?::14q32 → 14q32::?), +mar.ish der(5)(CMYC‐, IGH+, BCL2+), der(6)(CMYC‐, IGH+, BCL2+), ins(8;?)(CMYC+, IGH‐, BCL2‐), der(13)(CMYC‐I, GH+, BCL2‐), der(14)(CMYC+, IGH+, BCL2‐), der(?)(CMYC‐, IGH+, BCL2‐) [8 cells]/ 46, XY [12 cells]
22	65 M	Bone marrow	NA	NA	NA	45, XY, t(1;11;2)(q21;q11;p13), **t(8;22)(q24.1;q11.2)**, t(14;18)(q32;q21), ‐21 [14 cells]/46, XY [6 cells]
23	67F	Bone marrow	NA	5	Expired	46, XY, dup(1)(q42q21), **t(8;22)(q24;q11.2**), ‐12, t(14;18)(q32;q21), t(15;21)(p11.2;q11.2), +der(?)t(?;12)(?;q13) [21 cells]/46, idem, ‐dup(1) [3 cells]/47, idem, +der(8)t(8;22) (q24;q11.2) [4 cells]/46, XY [2 cells].nuc ish 8q24(CMYCx3), 14q32(IGHx3) [93 cells]/ 8q24(CMYCx4), 14q32(IGHx3) [66 cells]/8q24(CMYCx2), 14q32(IGHx3) [13 cells] 8q24(CMYCx2), 14q32(IGHx2) [28 cells]/14q32(IGHx3)(IGH con BCL2 × 2), 18q21(BCL2 × 3) [173 cells]/14q32(IGHx2), 18q21(BCL2 × 2) [27 cells]
24	59 M	Lymph node	NA	NA	NA	60, XY, +der(1;6)(q10;p10), +2, +del(3)(q12q21), +del(5)(q11.2q15), +7, +9, +11, +11, +15, +15, +18, +19, +add(19)(q12), +21[2]/59, idem, ‐2, +3, ‐del(3)(q12q21), **t(8;22)(q24;q11.2)**, ‐add(19)(q12), +add(19)(q12)[6]/45, X, ‐Y[4]/46, XY[8]
25	38 M	Bone marrow	Rituxan, hyperCVAD	13	Expired	47, XY, +der(1)del(1)(p34p36.1)inv(1)(p22p32), der(3)(3pter → p25::q27 → q11.2::p21 → q11.2::q27 → qter), **t(8;22)(q24.1;q11.2)**[20]
26	67 M	Bone marrow	R‐CHOP, hyperCVAD	8	Expired	39∼45, Y, del(X)(q22q28), add(1)(q32), add(2)(q31), add(3)(q12), t(7;12)(q22;q13), **t(8;22)(q24;q11.2)**, t(14;18)(q32;q21), add(16)(q12), add(17)(q21), ‐21, +mar[cp3]/46, XY[1]
27	78 M	Bone marrow	NA	NA	NA	70 < 3n > , XX, ‐Y, +add(1)(p13), der(1;14)(q10;q10), +der(1;16)(q10;p10)t(1;4)(q42;q21), add(2)(q37), ‐4, der(4)t(1;4), add(5)(q11.2), +7, **t(8;22)(q24.1;q11.2)**, **+**der(8)t(8;22), add(9)(q22), add(11)(p11.2), del(12)(q13q24.3), ‐13, i(13)(q10), +15, add(16)(q12.1), ‐18, +20, +add(21)(p12)[6]/45, X, ‐Y[3]/46, XY[10]
28	54 M	Peritoneal fluid	NA	1	Expired	91∼94 < 4N > , XXYY, der(1)t(1;13)(p13;q12), +der(1)t(1;13), +7, **t(8;22)(q24;q11)**, +12, ‐13, ‐18[cp6]/84∼86, idem, ‐der(1)t(1;13)x2, i(1)(q10), ‐4, ‐9, ‐10, ‐17[cp5]/73∼95, idem, ‐der(1)t(1;13)x2, idic(1)(p22), +5, ‐6, ‐10, ‐13, ‐16, ‐17[cp9].nuc ish(5′BCL6, 3′BCL6)x3∼5(5′BCL6 con 3′BCL6 × 3∼5)[144/200], (MYC x 5∼6, IGHx4)[162/200], (5′MYC, 3′MYC)x4(5′MYC sep 3′MYCx2)[123/200], (5′MYCx3∼4, 3′MYCx2∼3)(5′MYC sep 3′MYCx2∼3)[25/200], (IGHx3∼4, BCL2 × 3∼4)[169/200]
29	53 M	Retroperitoneal mass	R‐CHOP	7	Expired	43, XX, del(1)(q42q44), der(2)**t(2;8)(p13;q24)**, der(3)t(2;3)(p13;q27), 4, add(5)(q33), add(8)(q24), add(9)(q22), ‐10, add(10)(q24), ‐15, add(17)(p13).ish der(2)(CMYC+, IGH‐), add(8)(q24) (CMYC+, IGH‐) [13 cells].nuc ish 8q24(CMYCx1, CMYC spx1), 14q32(IGHx2) [121 cells]/8q24(CMYCx2, CMYC spx2), 14q32(IGHx6) [11 cells], 8q24(CMYCx2), 14q32(IGHx2) [68 cells]
30	63 M	Bone marrow	R‐CHOP, hyperCVAD	24	Expired	47∼48, X, ‐Y, add(1)(p32), add(1)(q32), der(2)**t(2;8)(q23;q24.1)**, ‐4, del(6)(q13q21), dic(6;20) (q13;q13.1), der(7)t(1;7)(q12;q36), add(9)(p22), add(9)(p12), +del(10)(p11.2p15), add(12) (q21), +13, i(18)(q10), +mar1, +mar2[cp2]/46∼54, idem, ‐4, ‐dic(6;20), +dic(6;8)(q13;q24.1), ‐del(10), +i(18), +20[cp2]/48∼49, idem, ‐add(1)(q32), +der(1)add(1)(p36.3)add(1)(q32), ‐der(2), +add(2)(q23), add(3)(p21), +add(4)(q21), ‐dic(6;20), +dic(6;8), del(7)(q32q36), ‐der(7)t(1;7), +der(7)t(1;7)(q12;q36)add(1)(q42), del(8)(p21p23), ‐add(9)(p22), +del(9)(p12p24), +dic(18;22) (p11.3;p12), +i(18), +del(20)(q11.2q13.3), ‐mar2, +mar3[cp3]/42∼48, idem, t(X;3)(q26;q21), del(4)(q12q21), der(5)t(1;5)(q12;q15), ‐dic(6;20), +dic(6;8), ‐der(7)t(1;7), +der(7)t(5;7) (q15;q36), ‐del(10), ‐add(12)(q21), +add(12)(q13), +i(18), +der(18;18)(q10;q10)t(10;18)(q11.2;q21), +20[cp8]/46, XY[5].ish der(2)t(2;8)(5′MYC‐, 3′MYC‐;5′MYC‐, 3′MYC+), dic(6;8)(5′MYC‐, 3′MYC‐;5′MYC+, 3′MYC‐)[15].nuc ish(5′BCL6, 3′BCL6)x2(5′BCL6 con 3′BCL6 × 2[200], (5′MYC, 3′MYC)x2 (5′MYC sep 3′MYCx1)[63/200], (CCND1, IGH)x2[200], (IGHx2, BCL2 × 5)[21/200]/(IGHx2, BCL2 × 7) [59/200]
31	43 M	Bone marrow	NA	NA	NA	46, Y, add(X)(p22.1), add(4)(q31), add(4)(q31), ins(7;?)(p13;?), t(8;10)(q21.2;q11.2), add(9)(p22), add(14)(q32), add(16)(p11.2), del(22)(q11.2q13.3) [3 cells]/47, idem, +4, ‐add(4)(q31), +r [3 cells]/46, XY [14 cells]
32	53 M	Tumor left flank	R‐CHOP, ESHAP‐R	21	Expired	41∼48, X, ‐Y, del(1)(q32q44), der(1)add(1)(p36.1)dup(1)(q21q32), ‐2, add(3)(p13), add(4)(p16), ‐6, der(6)t(6;14)(p23;q11.2)t(14;18)(q32;q21), der(7)add(7)(p13)add(7)(q32), ‐8, add(9)(q34), ‐10, ins(12;?)(q13;?), der(14)t(14;18)(q32;q21), ‐15, del(16)(q13q22), der(17)t(8;17)(q13;p13), ‐18, +der(?)t(?;1)(?;q25), +der(?)t(?;2)(?;q11.2), +der(?)t(?;18) (?;q11.2)t(14;18)(q32;q21), +r, +mar1, +2∼15mar [cp20 cells]nuc ish 14q32(IGHx3), 18q21 (BCL2 × 3)(IGH con BCL2 × 2) [166 cells]/14q32(IGHx3), 18q21(BCL2 × 4)(IGH con BCL2 × 2) [23 cells], 14q32(IGHx4), 18q21(BCL2 × 4)(IGH con BCL2 × 3) [11 cells]
33	58 M	Bone marrow	NA	NA	NA	48, X, ‐Y, +X, der(1)t(1;1)(p32;q12), del(6)(q13q21), +7, add(8)(p21), add(14)(q32), +der(?)t(?;1)(?;p22) [3 cells]/48, idem, add(16)(q22) [4 cells]/48, idem, t(10;19)(q22;p13.3), add(16)(q22) [4 cells]/46, XY [9 cells]
34	62 M	Bone marrow	NA	19	Expired	48, XY, +5, +12, add(14)(q32) [2 cells]/47, idem, ‐5, del(9)(p22p24), der(21)t(17;21)(q11.2;p11.2) [5 cells]/47, idem, add(1)(q42), ‐5, del(9)(p22p24), der(21)t(17;21)(q11.2;p11.2) [4 cells]/46, XY [9 cells] nuc ish 14q32(IGHx2), 18q21(BCL2 × 2) [200 cells]
35	67 M	Lymph node	NA	5	Expired	47, XY, +X, add(2)(p25), i(6)(p10), t(14;16)(p11.2;p11.2), add(17)(q25) [20 cells]
36	68 M	Bone marrow	NA	NA	NA	43, XY, del(1)(p13p22), ‐8, add(10)(q25), ‐13, ‐14, add(22)(p11.2) [3cells]/46, XX [27 cells] .nuc ish 13q14(RB1 × 1) [10 cells]/13q14(RB1 × 2) [190 cells]
37	47 M	Mediastinum thymus	NA	NA	NA	53, X, ‐Y, +X, der(4)add(4)(p16)dup(4)(q25q27), +5, add(12)(p13), +add(12)(p13), ‐15, add(16)(p13), +add(17)(q23), add(19)(q13.3), +21, +21, +21, +mar1, +mar2 [7 cells]/46, XY [10 cells]
38	52 M	Bone marrow	EPOCH	8	Expired	48, XY, +12, +14[2]/46, XY[18]
39	55 M	Groin testis	R‐CHOP, hyper‐CVAD	10	Expired	45, XY, t(2;11)(q23;q12), add(3)(p12), ‐9, ‐9, add(14)(q32), add(17)(p11.2), +mar[9]/46, XY[11]
40	55 M	Groin testis	CHOP, radiotherapy	63	Expired	48∼91 < 4n > XXY, +X, ‐4, +der(6)t(1;6)(q21;q13), del(9)(q22), add(12)(p11.2), del(12)(p13), add(13)(p11.2)x2, ‐14, add(14)(q32), der(16)t(12;16)(q13;p13.3)x2, ‐17, dup(18)(q21q22)x2, ‐19, +20, +1∼2rs
41	82F	Nasal mass	Anthracycline‐based chemotherapy (CDOP) plus Rituxan	93	Expired	42∼50, XX, der(1)del(1)(q32q44)dup(1)(q32q21), add(7)(p13), +del(7)(p13p15), del(15)(q15q22), +2r, +mar[cp15]
42	60 M	Lymph node	R‐CHOP	71	Expired	48, XY, +X, +3, add(7)(q32), add(10)(p11.2), der(15;21)(q10;q10), i(17)(q10), +18[9]/46, XY[9] (lymph node)
43	51 M	Lymph node	RESHAP with prophylactic intrathecal chemotherapy with methotrexate	74	Alive	53, Y, add(X)(p22.1), +5, +8, del(8)(q11.2q24.1)x2, +12, del(13)(q12q14), t(14;18)(q32;q21), +18, +20, +21, +22[cp15]/54, idem, +6[4]/46, XY[1].nucish(MYCx3, IGHx2)[33/200]/(MYCx4, IGHx2)[112/200]/(MYCx4, IGHx3)[26/200], (IGHx3, BCL2 × 4)(IGH con BCL2 × 2)[182/200]
44	80 M	Bone marrow	NA	1	Expired	48, XY, t(1;9)(p34;p22), trp(1)(q32q42), add(4)(q35), +8, add(22)(q13), +mar[13]/46, XY[7] (bone marrow)
45	60 M	Thyroid mass	R‐CHOP	97	Alive	47, X, ‐Y, add(1)(q21), der(1)t(1;3)(p13;q27)add(1)(q32), der(3)t(1;3)del(1)(p34p36.1), add(11)(q23), +16, +21[20]
46	50 M	Bone marrow	CHOP‐ Rituxan not given in the setting of CD4 <200)	1	Expired	49, XY, +X, dup(1)(q11q42), add(2)(q11.2), add(4)(p16), del(4)(q12q21), ‐5, add(8)(q24.3), del(8)(q24.1q24.3), ins(14;?)(q24;?), del(15)(q11.2q22), del(16)(q22q24), del(18)(p11.1p11.2), add(19)(p12), +der(?)t(?;5)(?;q11.2), +mar1, +mar2[3]/46, XY[19]
47	67F	Lymph node	R‐CHOP, 2X RICE, SCT	152	Alive	46, X, ‐X, +3, der(14)t(14;18)(q32.3;q11.2), add(17)(p13), dup(18)(q21q23), der(21)t(11;21)(q13;p12) [18]/46, XX[2].nuc ish(5′BCL6, 3′BCL6)x3(5′BCL6 con 3′BCL6 × 3)[187/200], (5′MYC, 3′MYC)x2 (5′MYC con 3′MYCx2)[200], (IGHx2, BCL2 × 4)[83/200]/(IGHx2, BCL2 × 5)[102/200]
48	60 M	Basal ganglia lesion	R CHOP and IT MTX	14	Expired	40∼49, XY, der(1)t(1;3)(q23;q27), der(3)t(3;7)(p21;q32)t(1;3), 4, add(4)(p14), +5, del(6)(p23p25), der(7)t(3;7), +9, +12, +13, der(14;17)(q10;q10), 16, +add(17)(q25), add(19)(q13.4), +mar[cp9]/46, XY[1]
49	66F	Bone marrow	R‐CHOP	22	Expired	44∼47, XX, +X, add(1)(p34), add(1)(q21), add(2)(p11.2), add(3)(q11.1), add(3)(q11.2), ‐4, ‐6, i(6)(p10), +add(9)(q34), add(11)(q23), add(13)(p11.2), der(14)t(14;18)(q32;q21), der(15;17) (q10;q10), add(16)(q22), +18, ider(18)(q10)t(14;18), der(21)t(3;21)(q12;p11.2), add(22)(q13), +1∼2mar[cp4]/87, idemx2[1]/46, XX[15].nucish(ATMx2)[200], (D12Z3 × 2)[200], (RB1 × 2)[200], (TP53 × 2)[194]
50	73 M	Lymph node	R‐CHPOP	52	Expired	54∼57, XY, +X, +der(1;9)(q10;q10), +del(5)(q13q33), +10, +12, t(14;18)(q32;q21), add(15)(q22), +21, del(22)(q11.2q13), +4∼5mar[cp6]/46, XY[14]
51	41 M	Peripheral blood	NA	NA	NA	46∼47, XY, der(1)add(1)(p36.1)dup(1)(q12q35), add(14)(q32), t(14;18)(q32;q21), +21[cp4]/46, XY[16]
52	51 M	Lymph node	Cytarabine, Rituxan with IT chemotherapyR‐EPOCH	27	Alive	46, XY, add(1)(q32), t(2;12)(p11.2;p13), add(4)(p16), add(5)(p15), add(10)(p11.2), add(11)(p15), ‐13, add(17)(p11.2), +mar[cp3]/46, XY[17]
53	54 M	Lymph node	NA	NA	NA	58∼73 < 3n > , XY, add(3)(q12), add(5)(q31), i(6)(p10), dup(7)(q11.2q32), add(9)(p13)x2, +11, del(11)(q21q25)x2, ‐12, ‐13, ‐14, ‐14, ‐15, ‐15, add(15)(p12), ‐16, ‐19, add(19)(p13), ‐21, ‐21, ‐22, ‐22, +12∼21 mar[cp6]/46, XY[1]
54	23F	Groin mass	DA‐R‐EPOCH	24	Alive	47∼48, XX, der(3)t(3;8;14)(q27;q24;q32)add(14)(q32), +7, der(8)t(3;8;14), der(8)dup(8)(q22q24) add(8)(q24), +12, add(14)(q32), der(14)t(3;8;14)del(14)(q24q31)[19]/46, XX[1].ish der(3)(3′BCL6+, 5′BCL6‐, IGH+), der(8)**t(3;8;14**)(3′BCL6‐, 5′BCL6+, 5′MYC+, 3′MYC‐, IGH‐), der(8)dup(8)(MYC++), der(14)(3′BCL‐, 5′BCL6‐, 5′MYC‐, 3′MYC+, IGH+)[cp9].nuc ish(5′BCL6, 3′BCL6)x2(5′BCL6 sep 3′BCL6 × 1)[141/200], (MYCx3∼4, IGHx3∼6)(MYC con IGHx1∼2)[122/200], (5′MYC, 3′MYC)x2∼3(5′MYC sep 3′MYCx1∼2)[129/200], (IGHx3, BCL2 × 2) [110/200]
55	63F	Lymph node	NA	4	Expired	44, X, ‐X, add(1)(q32), dup(3)(q11.2q29), ‐4, del(5)(p14p15), add(6)(q23), del(6)(q13q27), +7, add(10)(p13), i(11)(q10), der(12;17)(p10;q10), add(14)(p11.2), ‐15, +18[cp2]/44, idem, der(4)t(3;4)(q11.2;p14)[cp14]/44∼45, idem, +3, +add(3)(p21), ‐add(6), +6[cp4]
56	40 M	Lymph node	R‐CHOP, CAR‐T	17	Alive	48, XY, +X, +Y, t(2;22)(p13;q11.2), del(4)(q12q25), der(5)t(4;5)(q25;p15), add(9)(p24), add(9)(p22), add(11)(q23), add(12)(q24.1)[cp10]/52, idem, +7, +13, +15, +18[cp2]/46, XX[1]
57	66 M	Lymph node	R‐CHOP	18	Alive	49, XY, +7, +12, t(14;18)(q32;q21), 15, +add(19)(q13.3), +21, der(22)t(15;22)(q15;q13)[cp14]/49, idem, der(14)t(14;18), der(18)add(18)(p13)t(14;18)[cp5]/46, XY[1]
58	63 M	Lymph node	R‐CHOP, RICE, fludarabine and cyclophosphamide for CART	18	Expired	73∼81 < 4n > , XX, ‐Y, ‐Y, der(1)add(1)(p13)t(1;7)(q32;q22)x2, der(1;17)(q10;q10)x2, add(2)(q13), ‐3, ‐3, ‐4, add(4)(q31), ‐5, der(6)t(3;6)(p11;q13)x2, add(7)(q36)x2, ‐9, add(9)(q34)x2, ‐10, der(11)add(11)(p11.2) add(11)(q23)x2, add(12)(q15), ‐16, ‐17, ‐17, del(22)(q12q13), +1∼5mar[cp7]/80∼81, idem, i(6)(q10)[cp2]/ 76∼80, idem, i(6), add(9)(p24)[cp4]
59	84 M	NA	NA	NA	NA	51, X, ‐Y, +2, +4, +7, t(8;14)(q24;q32), t(10;20)(q21;q13.1), +18, +r, +mar[19]/47, XY, +Y[1]

Abbreviation: ID, identification number; NA, not available; R‐CHOP, rituximab, cyclophosphamide, doxorubicin hydrochloride, vincristine, and prednisone; DA, daunorubicin, Ara‐cytarabine; IT MTX, intratechal methotrexate; RICE, rituximab, ifosfamide, carboplatin, etoposide; SCT, stem cell transplant; CDOP, cyclophosphamide, doxorubicin, vincristine, prednisone; EPOCH, etoposide, prednisone, vincristine, cyclophosphomide, doxorubicin.

**FIGURE 9:**
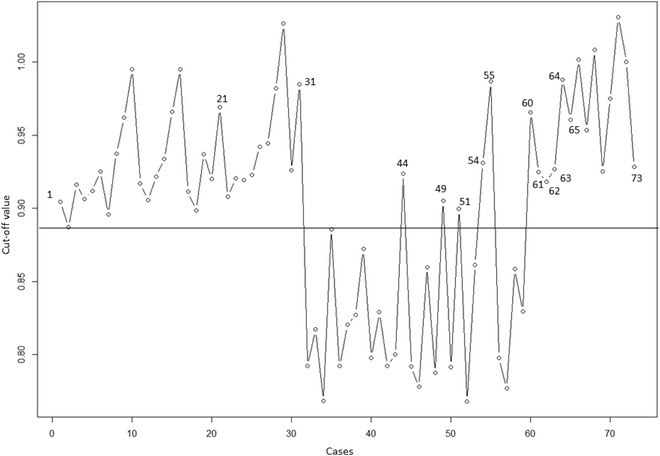
Classification of cohort 2 cases and 14 cases with nonclassic *MYC*+ due to complex translocations involving *MYC* and *MYC* nonimmunoglobulin (*IG*) cases from cohort 1 by the NNET artificial intelligence algorithm. Receiver operating characteristic curve cut‐off value of 0.89 is considered as discriminator between *MYC*+ and *MYC*− cases (i.e, optimal specificity and sensitivity). Cases above the horizontal line are classified as *MYC*+ and cases below the horizontal line are *MYC*‐. Cases 44, 49, 51, and 55 (from cohort 1) are *MYC*− by chromosomes and FISH but had higher genetic progression score and the AI algorithm recognized them as *MYC*+

When predicting the *MYC* status in the 59 institutional cases, the NNET AI algorithm correctly classified 55 cases at a cut‐off value of 0.89 from the ROC curve. This algorithm correctly classified all *MYC*+ tumor cases (cases 1–31 from cohort 2) including the two cases with cryptic rearrangement (cases 21 and 54), but misclassified four MYC− cases as *MYC*+ (cases 44, 49, 51, and 55) because these had elevated GPS. The specificity and sensitivity of this algorithm was 87% and 100% each at predicting *MYC* status with a positive predictive value of 92% and a negative predictive value of 100%. Since cohort 2 had only two cases (case 21 and 54) with a cryptic *MYC* R, we mixed five cryptic *MYC*+ cases (cases 60–64) and nine *MYC* non‐*IG* cases (cases 65–73—inv(8)(p21q24), t(4;8)(q21;q24), t(8;18)(q24), t(3;8)(q27;q24)x2, t(3;8)(p24;q24)x2, t(8;9)(q24;p13) and t(8;16)(q24;p11)) from cohort 1 with cohort 2 cases and then applied the AI algorithm. All these cases were correctly assigned to expected group (Figure 9). Thus, the AI algorithm correctly classified classical *MYC* R [t(8;14), t(8;22), t(2;8)], cryptic complex *MYC*, and *MYC*/non‐*IG* rearrangement with high fidelity. However, given the small dataset, additional MYC/non‐IG cases or cryptic MYC+ cases are warranted to test the robustness of this algorithm.

**FIGURE 10:**
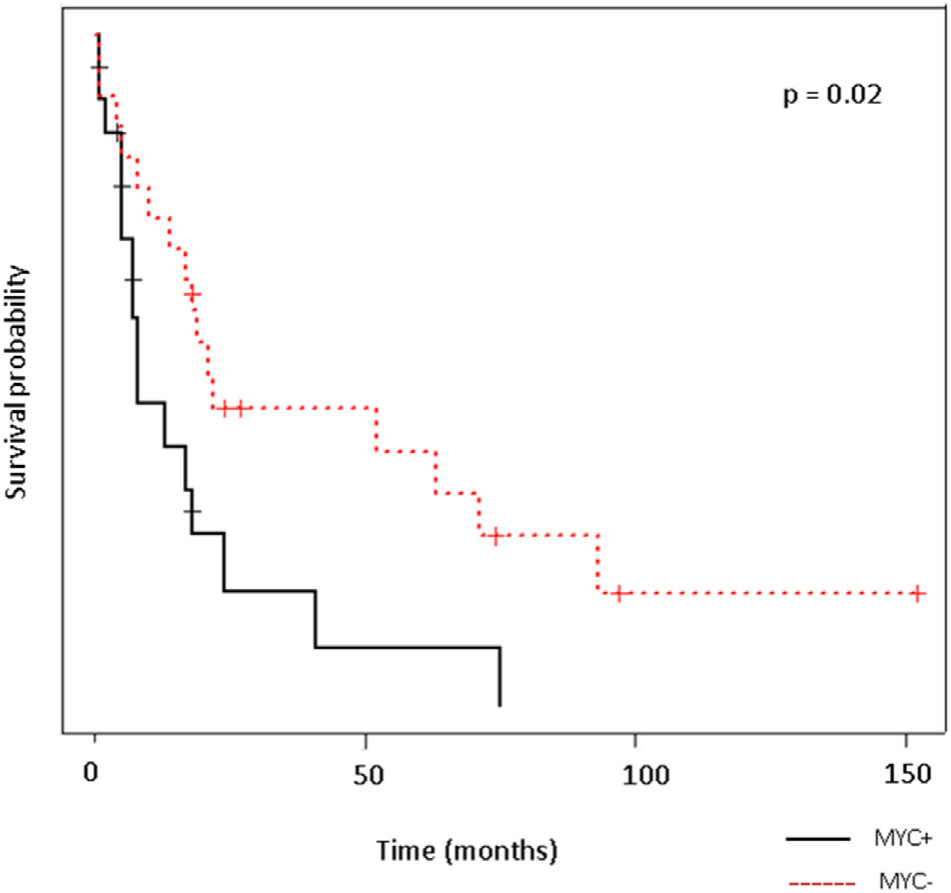
Kaplan–Meier and log rank survival test for tumors with and without a *MYC* rearrangement in cohort 2 cases. *MYC*+ tumors had significantly lowered survival

Clinical outcome was available for 44 patients in cohort 2. In agreement with the literature, cases with a *MYC* R showed a shorter survival (Figure 10).

## 3 DISCUSSION

*MYC*+ DLBCL has poor clinical outcome compared to *MYC−* DLBCL. For example, when treated with CHOP‐like and augmented CHOP‐like therapies, the 5‐year survival in *MYC*+ DLBCL patients was inferior compared to *MYC−* patients (44% vs. 67%; *p* = 0.001) [7]. Even when treated with rituximab and anthracycline‐based therapies, *MYC*+ DLBCL maintained a poor clinical outcome [1, 9]. In pediatric patients, event free survival was six‐fold less in *MYC*+ cases compared to *MYC−* [28]. Likewise, in the germinal center (GC)‐DLBCL that carries a favorable prognosis, *MYC*+ negates the positive outcome [11]. Therefore, detection of such rearrangements is of clinical importance.

At present, methods used in a clinical setting to detect *MYC* abnormalities include chromosome analysis and FISH. Although chromosome and FISH analysis detects *MYC* R, these techniques may lack the specificity to detect all cases. For example, chromosome analysis lacks the resolution to detect cryptic rearrangements involving *MYC* and *IG* loci, while FISH analysis using both the dual color dual fusion and break‐apart probe approaches may not detect all *MYC* abnormalities due to the large variation of *MYC* breakpoints. Although next generation sequencing (NGS) is widely implemented in clinical diagnosis and has ability to detect structural rearrangements such as translocations, it suffers similar shortcomings of FISH in detecting *MYC* status due to variation in breakpoints. Indeed, variation of breakpoints has been reported well outside the 5′ and 3′ ends of *MYC* [21]. In terms of translational oncology (IHC), as of now, there is no immuno‐phenotypic marker that can distinguish a *MYC*+ versus a *MYC−* DLBCL. To address this issue and find screening tests for *MYC* R, Rodig et al. [29] reported using VpreB3 expression detected by IHC to predict *MYC* gene aberrations; however, the antibody used in this study is not widely available. In terms of molecular techniques, long distance polymerase chain reaction (PCR) using nested PCR with the use of specific breakpoint primers has been used to detect minimal residual disease in patients with *MYC‐IGH* rearrangement [30, 31]; however, primer design may not be optimal to detect all *MYC* breakpoints in newly diagnosed cases. Other more complex technique such as chromatin immunoprecipitation with subsequent deep sequencing has been developed to map 7054 *MYC‐IGH* binding sites [32], although validation of this data is required, and this technique is out of reach to clinical settings. Another recently reported method for detecting chromosome rearrangements is translocation capture sequencing [33, 34], but like the approach just mentioned, it remains mainly in research settings. In contrast to these methods, new translational models in the form of AI algorithm may further enhance our diagnostic ability. Indeed, such systems have been proven useful for screening B‐cell lymphomas using deep learning methods with convolutional neural networks, digital microscopic imaging, and the use of AI algorithm to predict the prognosis of DLBCL patients [35, 36, 37]. In this context, application of AI algorithm applied to cytogenetic data would be greatly beneficial. Therefore, in this analysis, we explored whether an AI algorithm composed of RCAs and GPS can assess *MYC* status in the karyotypes of DLBCL cases from the literature, then validated its ability to predict *MYC* status in our institutional cases, and demonstrated that properly developed AI algorithm can predict *MYC* status in these tumors.

Evaluation of RCAs revealed gain of chromosome 1p and band regions 1q10‐q32 significantly more prevalent in *MYC*+ tumors, while chromosome losses were more prevalent in *MYC−* tumors. Using six additional classifiers from the azure machine learning Microsoft platform, GPS was recognized as the most important predictor of *MYC* status. When assessing driver alterations, *MYC* was the sole driver aberration in *MYC*+ tumors, and evolution patterns in these tumors revealed +3, and losses to 6p21, 7p15, and 17q23 correlated with *MYC* proliferation expression profiles and IHC (mainly *FOXP1*, *MYD88*, *PIM1*, *CARD11*, and *CD79B* mutations) [38]. We also observed gains of 13, 15, 16, 20, and loss of 17 and 19 late in disease progression of *MYC*+ tumor cases. A significant difference in the number of alterations and type of RCAs in *MYC*+ versus *MYC−* tumors, represented by a higher GPS value in *MYC*+ tumor cases, was also documented. In the context of AI, various models predicted *MYC* status with high fidelity, both for classical and nonclassical rearrangements. However, confirmation of these results in larger dataset of *MYC*/non‐*IG*, and cryptic *MYC* R is warranted.

Clinical significance of *MYC* R, other than in double hit cases, has not been established. A few studies examined the biological significance of concurrent non‐classical *MYC* and *BCL2* gene rearrangements in DLBCL patients. Hilton et al. showed that genetic signature of these patients is similar to typical DHIT DLBCL patients [23]. Li et al. evaluated the clinico‐pathological features of DLBCL patients with concurrent atypical *MYC* and BCL2 rearrangements and compared with DLBCL patients with typical DHITs and found that overall survival between these two groups was similar [39]. Sweden et al. [40] showed that clinical outcome in patients with *MYC*/*IG* rearrangement was inferior to patients with *MYC*/non‐*IG* R. Dose‐adjusted intensive treatment with EPOCH‐R in patients with *MYC*R showed promising outcome [13]; another study using lenalidomide and R‐CHOP showed positive outcome in *MYC+* patients [41]. Although the frequency of *MYC*/non‐*IG* or cryptic *MYC*+ is high in *MYC*+ DLBCL [42], studies on whether DLBCL tumors with non‐*IG* and cryptic *MYC* R protend clinical features akin to classical *MYC* R are very limited. Additional studies that focus on *MYC*/*IG*, *MYC*/non‐*IG*, and cryptic *MYC* R to assess differences in survival and whether these groups share similar genetic profiles are warranted.

Although our observations are preliminary, we showed in this proof‐of‐concept study that properly built and validated AI algorithm can reliably detect *MYC* status in these tumors and paves the way for future studies in applying AI algorithm for predicting cytogenetic alterations of clinical interest. We recognize the paucity of fresh data from our institutional cases, and we anticipate that larger datasets (from larger centers or multi‐center collaborations) are required to validate these results. We also acknowledge that this is a retrospective study and were unable to further evaluate all *MYC* negative cases with a dual color dual fusion and break apart approach to assess undetectable *MYC* R in metaphase cells. Likewise, prospective studies may benefit from using mitogens such as lipopolysaccharide, cytokines, or oligonucleotides to stimulate tumor cells in culture to capture metaphases of all abnormal cells to rule out a selection bias. We also recognize tumor samples presenting with a simple karyotype (i.e., a *MYC* R with one or two additional aberrations) may not be suitable for AI; however, we should highlight that DLBCL and large B‐cell lymphomas with *MYC* R often present with complex karyotypes. Additional research to develop built‐in models via web‐portals or computer software programs to calculate GPS and leverage AI algorithm based on cytogenetic data analysis is warranted to further enhance our diagnostic and prognostic accuracy in the cytogenetic laboratory.

## FUNDING INFORMATION

The authors received no specific funding for this work.

## CONFLICT OF INTEREST

The authors declare they have no conflicts of interest.

## ETHICS STATEMENT

This work reflects the authors’ own research and analysis.
